# Smooth Muscle Cell Responses to Poly(ε-Caprolactone) Triacrylate Networks with Different Crosslinking Time

**DOI:** 10.3390/ijms21238932

**Published:** 2020-11-25

**Authors:** Jing Wang, Li Liu, Aoning Wang, Xiang Liu, Yi Zhang, Zhoulu Wang, Jinbo Dou

**Affiliations:** 1 School of Energy Sciences and Engineering, Nanjing Tech University, Nanjing 211816, China; wjx2015@njtech.edu.cn (J.W.); iamxliu@njtech.edu.cn (X.L.); 2 Key Laboratory of Flexible Electronics (KLOFE), Institute of Advanced Materials (IAM), Nanjing Tech University, Nanjing 211816, China; 201861122099@njtech.edu.cn (L.L.); aoningsmile@gmail.com (A.W.); 3 Department of Materials Science and Engineering, The University of Tennessee, Knoxville, TN 37996, USA

**Keywords:** poly(ε-caprolactone) acrylates, crosslinking time, mechanical properties, smooth muscle cells

## Abstract

Poly(ε-caprolactone) triacrylate (PCLTA) is attractive in tissue engineering because of its good biocompatibility and processability. The crosslinking time strongly influences PCLTAs cellular behaviors. To investigate these influences, PCLTAs with different molecular weights were crosslinked under UV light for times ranging from 1 to 20 min. The crosslinking efficiency of PCLTA increased with decreasing the molecular weight and increasing crosslinking time which could increase the gel fraction and network stiffness and decrease the swelling ratio. Then, the PCLTA networks crosslinked for different time were used as substrates for culturing rat aortic smooth muscle cells (SMCs). SMC attachment and proliferation all increased when the PCLTA molecular weight increased from 8k to 10k and then to 20k at the same crosslinking time. For the same PCLTA, SMC attachment, proliferation, and focal adhesions increased with increasing the crosslinking time, in particular, between the substrates crosslinked for less than 3 min and longer than 5 min. This work will provide a good experimental basis for the application of PCLTA.

## 1. Introduction

Poly(ε-caprolactone) (PCL) is a biodegradable semi-crystalline polymer that has been widely used in tissue engineering [1,2,3,4,5]. The crystalline structure offers PCL unique thermal and mechanical properties. After the hydroxyl end groups in PCL diols or triols are converted into crosslinkable functional groups, i.e., acrylate, maleic anhydride, or fumarate, they can be crosslinked into networks with controllable chemical and physical properties [6,7,8,9,10,11,12]. Compared to the other crosslinking methods through heat, irradiation, or redox process, photo-crosslinking is more efficient to control the polymerization heat evolution and fabricate complex structures [13,14,15,16]. Wang et al. have developed photo-crosslinkable PCL acrylates (PCLAs) such as PCL diacrylates (PCLDAs) and PCL triacrylates (PCLTAs) [17]. Depending on the molecular weight of PCL precursor in the synthesis of PCLA, amorphous and semi-crystalline PCL networks were prepared with elastic modulus (*E*) ranging from 1 to 200 MPa and controllable melting temperature (T_m_) and crystallinity (χ_c_).

The crosslinks in the photo-crosslinked PCLAs suppressed and impeded the PCL crystallization, resulting in a reduced crystallinity. For photo-crosslinkable polymers, the crosslinking efficiency can be controlled by the density of crosslinkable groups, the concentration of photo-initiator, UV light intensity, and crosslinking time [18,19,20,21,22]. Among all these factors, sufficient crosslinking time should be given to ensure a polymer network with a high gel fraction and desirable mechanical properties without causing overcure and photo-scission problems [23,24]. For PCLAs, both crosslinking density and efficiency increase with increasing the density of acrylate end groups, which can be achieved by either decreasing the molecular weight of the PCL precursor with a certain architecture, or having multiple arms with more acrylate end groups in one polymer.

In this study, we studied the roles of crosslinking density and crystallinity in determining the physical properties, such as gel fraction, swelling ratio, and thermal, rheological, and mechanical properties, of photo-crosslinked PCLTA by varying both the crosslinking time and the molecular weight of the precursor. These PCLTA networks resulted from different crosslinking time were further used as substrates to regulate rat aortic smooth muscle cell (SMC) cell behaviors, including attachment, proliferation, spreading, and focal adhesion. This study demonstrates that photo-crosslinked PCLTA can not only promote SMC attachment, but also maintain focal adhesions, providing a new experimental scheme for the design and optimization of vascular tissue engineering scaffolds.

## 2. Results and Discussion

### 2.1. Gel Fraction and Swelling Ratio

**Figure 1:**
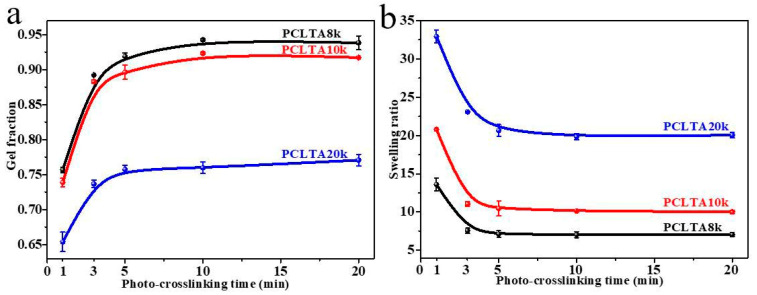
Gel fractions (**a**) and swelling ratios (**b**) of PCLTAs with different crosslinking time.

Crosslinking kinetics is important for understanding how a polymer network is formed and when crosslinking is sufficient for a variety of properties. The gel fraction and swelling ratio in the CH_2_Cl_2_ of photo-crosslinked networks of the PCLTAs with different crosslinking times are shown in Figure 1. Because the composition of acrylate groups was lower in PCLTA with a higher molecular weight, the gel fraction decreased from PCLTA8k to PCLTA10k and then PCLTA20k, at the same crosslinking time. For the same PCLTA, the gel fraction increased asymptotically with increasing the crosslinking time. The distance between two neighboring crosslinks in the network was determined by both the molecular weight of the PCL precursor and crosslinking time [14,17,19]. Therefore, the swelling ratios increased from the range of 7.0–13.5 for PCLTA8k networks to 9.8–21.0 for PCLTA10k networks, and then, 17.5–32.8 for PCLTA20k networks. For the same PCLTA, the swelling ratio of the network decreased with increasing the crosslinking time. The crosslinking efficiency was higher for PCLTA8k and PCLTA10k than for PCLTA20k. A crosslinking time of 10 min was needed to reach the highest gel fractions of 94% for PCLTA8k and 92% for PCLTA10k, while a longer crosslinking time of 40 min was needed for PCLTA20k to reach the highest gel fractions of 80%. When the crosslinking time increased from 1 to 3 min, the gel fraction increased and the swelling ratio decreased dramatically for all the PCLTAs with different molecular weights. When the crosslinking time was longer than 10 min, the increase in the gel fraction and the decrease in the swelling ratio of PCLTA8k and PCLTA10k could be barely noticeable, while the gel fraction increased and the swelling ratio decreased for PCLTA20k less sharply than the first 3 min.

### 2.2. Thermal and Mechanical Properties

**Figure 2:**
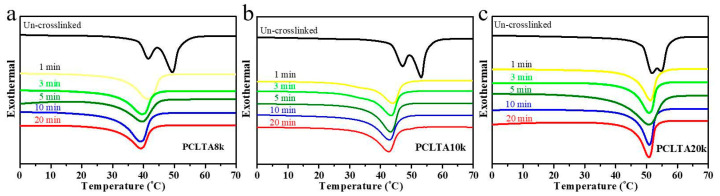
Differential scanning calorimeter (DSC) curves of crosslinked PCLTA8k (**a**), PCLTA10k (**b**), and PCLTA20k (**c**) with different crosslinking time.

The thermal properties (T_m_, ΔH_m_, and χ_c_) of photo-crosslinked PCLTAs for various crosslinking times in Table 1 were obtained from the DSC curves in Figure 2. T_m_ was the highest temperature of exothermal peaks during the heating round. χ_c_ was calculated using the equation of χ_c_ = [ΔH_m_/(Φ_PCL_∙ΔH_m_^c^)] × 100%, where ΔH_m_^c^ of completely crystalline PCL is 135 J/g [20]. All uncrosslinked PCLTAs were semi-crystalline with higher T_m_ and χ_c_ than the crosslinked counterparts as the crosslinks suppressed crystallinity. Nevertheless, all the crosslinked PCLTAs were still semi-crystalline at the physiological temperature (37 °C). At the same crosslinking time, both T_m_ and χ_c_ increased from PCLTA8k and PCLTA10k to PCLTA20k. The T_m_ of PCLTA10k was higher than that of PCLTA8k, while the difference in χ_c_ between PCLTA8k and PCLTA10k was insignificant. For the same PCLTA, T_m_ and χ_c_ decreased dramatically when the crosslinking time increased from 0 to 3 min. With increasing the crosslinking time further, χ_c_ still decreased, but at a much lower rate, while T_m_ only decreased slightly.

**Figure 3:**
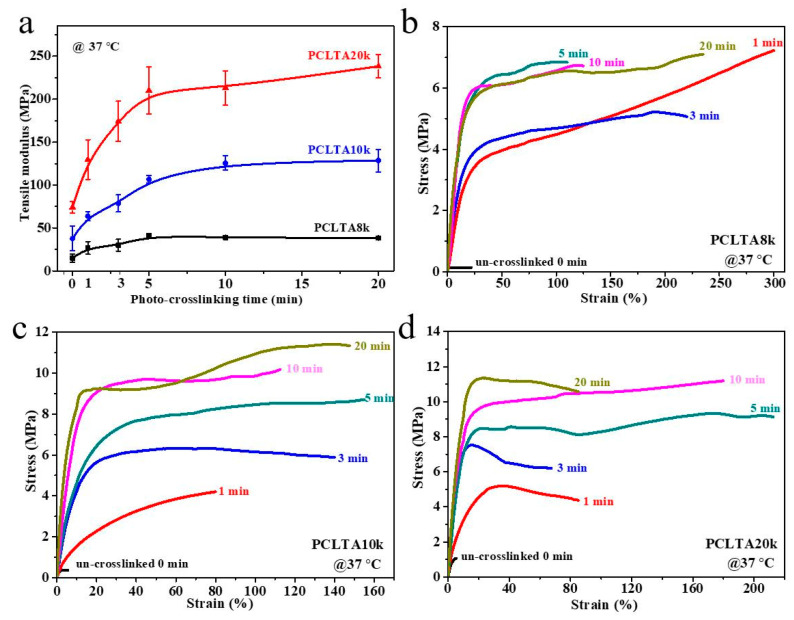
(**a**) Tensile modulus of PCLTAs (8k, 10k, and 20k) with different crosslinking time. Tensile stress–strain curves of PCLTAs ((**b**), 8k; (**c**), 10k; (**d**), 20k) with different crosslinking time.

Tensile testing was performed at 37 °C to obtain the mechanical properties of photo-crosslinked PCLTAs at various crosslinking times. Representative stress–strain curves are shown in Figure 3. The *E* increased from the range of 15.1–38.4 MPa for PCLTA8k to 37.9–128.5 MPa for PCLTA10k, and then 74.1–238.3 MPa for PCLTA20k. Because PCLTA20k had a higher χ_c_ than those of PCLTA8k and PCLTA10k, the stiffness increased with the increase of molecular weight at the same crosslinking time. Different from crosslinked PCLTAs, uncrosslinked PCLTAs were extremely brittle, with the strain at break of less than 10%. For the same PCLTA, the stiffness increased dramatically with increasing the crosslinking time from 0 to 5 min (15.1 to 41.0 MPa for PCLTA8k, 37.9 to 106.6 MPa for PCLTA10k, and 74.1 to 210.1 MPa for PCLTA20k), while the variance in stiffness was insignificant when the crosslinking time was longer than 10 min (41.0 to 38.4 MPa for PCLTA8k, 106.6 to 128.5 MPa for PCLTA10k, and 210.1 to 238.3 MPa for PCLTA20k). The stiffness of crosslinked PCLTA20k always increased with increasing the crosslinking time, while the highest stiffness of crosslinked PCLTA8k and 10k was observed at the crosslinking time of 5 and 10 min, respectively.

**Figure 4:**
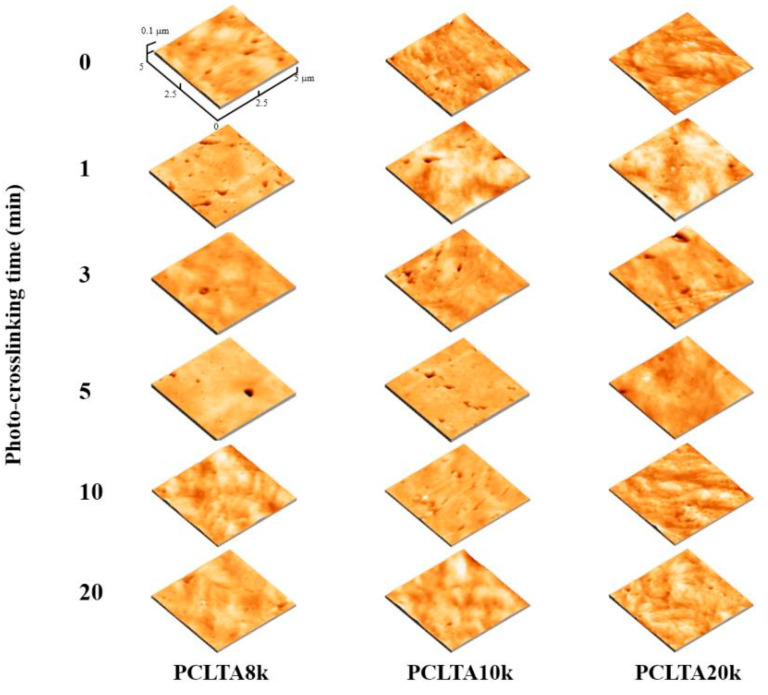
Atomic force microscope (AFM) 3D height images of PCLTAs with different crosslinking time.

Similar to uncrosslinked PCLTAs, crosslinked PCLTA disks at various crosslinking times were compressed and smoothened between two glass plates, and the surface morphologies were detected using AFM (Figure 4). After the compression, the *R*_rms_ values of all the polymer disks were less than 20 nm without a significant difference. Therefore, the effect of surface roughness on cell behaviors could be neglected.

### 2.3. In Vitro SMC Attachment and Proliferation

**Figure 5:**
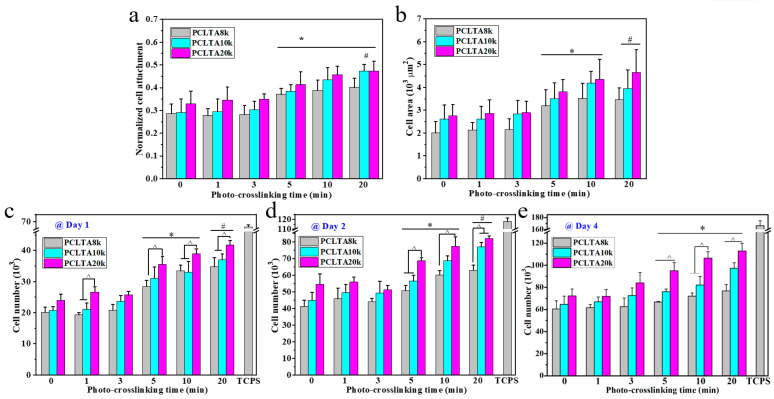
(**a**) Normalized smooth muscle cells (SMC) attachment at 4 h post seeding. (**b**) SMC areas at day 1 post-seeding. (**c**–**e**) SMC numbers at days 1, 2, and 4 post-seeding on the PCLTAs with different crosslinking time, respectively. *: *p* < 0.05 between the samples marked with the symbol with the corresponding data on the same PCLTA with crosslinking time of 0, 1, and 3 min. ^#^: *p* < 0.05 between the samples marked with the symbol with the corresponding data on the same PCLTA with crosslinking time of 0, 1, 3, and 5 min. ^^^: *p* < 0.05 between the samples marked with the symbol.

**Figure 6:**
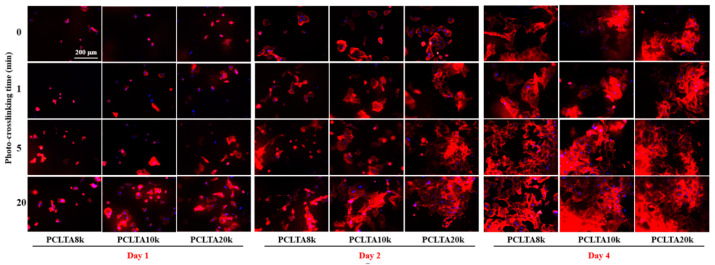
SMC images at days 1, 2, and 4 post-seeding stained using rhodamine-phalloidin (RP; red) and 4′,6-diamidino-2-phenylindole (DAPI; blue) on PCLTAs with different crosslinking time. Scale bar of 200 μm is applicable to all.

SMCs were seeded on the crosslinked PCLTA disks at various crosslinking times, and the results of cell attachment, proliferation, and area are shown in Figure 5. Consistent with the findings in previous studies [25,26,27,28], stiffer substrates could support SMC attachment and proliferation better, indicated by those on photo-crosslinked PCLTA20k were better than on photo-crosslinked PCLTA8k and PCLTA10k at the same crosslinking time. Specifically, the difference in SMC proliferation became more significant when the crosslinking time was longer than 5 min. For the same PCLTA, SMC attachment and proliferation were better when the crosslinking time was longer. Significant differences were found between the PCLTAs at crosslinking time longer than 10 min and shorter than 5 min. Among the crosslinking time of 0, 1, 5, and 20 min, SMC attachment and proliferation had no significant difference on the same PCLTA networks. The cell images stained with RP and DAPI (Figure 6) were consistent with the cell numbers obtained using the MTS assay. SMC spread areas also followed the same trend as in cell attachment and proliferation. However, no significant difference was found on different PCLTAs at the same crosslinking time. For the same PCLTA, when the crosslinking time was higher than 5 min, the increase in the cell area became more significant than on those at crosslinking time of 0, 1 min.

### 2.4. Focal Adhesions

**Figure 7:**
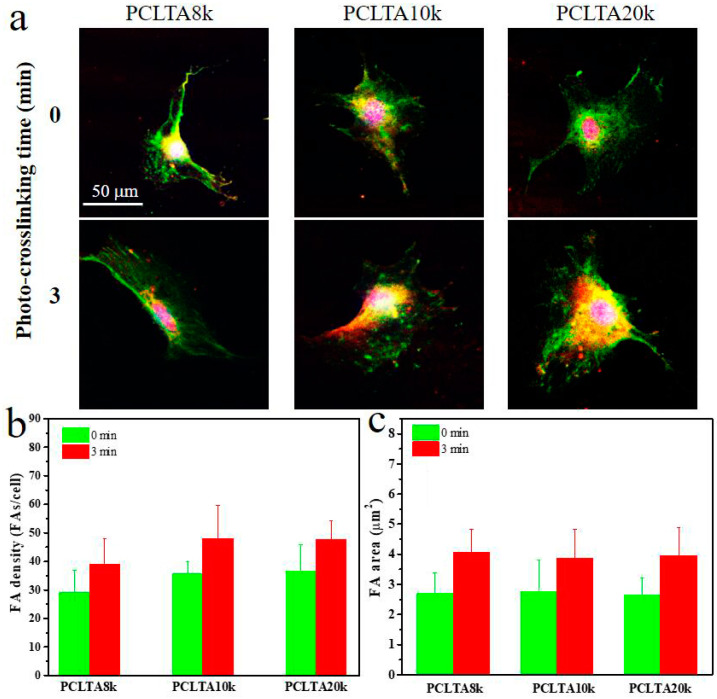
Characterizations of focal adhesions (FAs) in SMCs at day 1 post-seeding on PCLTAs with different crosslinking time (0, 3 min). (**a**) Immunofluorescence images of FAs in the cells with vinculin stained green, and F-actin stained red. Scale bar of 50 μm is applicable to all. Quantification of FAs in terms of (**b**) FA density, and (**c**) FA area.

FAs are able to respond to external mechanical signals and adjust their own properties and simultaneously trigger mechanotransduction for further regulation of cell growth and spreading, and therefore, were used to further evaluate cell adhesion to the polymer substrates. FAs in the SMCs cultured for one day on the crosslinked PCLTA8k, 10k, and 20k disks at crosslinking time of 0, and 3 min were characterized and the results are shown in Figure 7. The FA density and area had the same trend as in the cell attachment and proliferation. For the same PCLTA, both FA density and area increased when the crosslinking time increased from 0 to 3 min. At the same crosslinking time, FA density and FA area increased from PCLTA8k to PCLTA10k and then PCLTA20k; however, the difference was not significant among all the crosslinked PCLTAs at different crosslinking times.

### 2.5. Further Discussion

The present study attempted to understand how to achieve various thermal and mechanical properties through controlled crystallinity and crosslinking density by using PCLA networks that were crosslinked for different times. Without showing detectable cytotoxicity, all the PCLTA networks could support SMC adhesion, proliferation, and spreading. Meanwhile, the molecular weight and crosslinking density of PCLTAs resulted in different physical properties and, consequently, distinct SMC behaviors. Upon receiving the signals of the substrate materials, cells respond by adjusting their spreading, proliferation, and other related processes [29].

In terms of crosslinking efficiency, a difference was found between PCLTA20k and the other two PCLTAs with lower molecular weights. For PCLTA8k and PCLTA10k, crosslinking almost finished within 10 min of UV exposure, while for PCLTA20k, longer crosslinking time of 60 min could still increase the gel fraction and decrease the swelling ratio. The cell results showed the same trend with the gel fraction. With crosslinking time over 10 min, the densities of SMCs on crosslinked PCLTA8k and PCLTA10k disks did not vary too much. In contrast, the cell density on PCLTA20k still increased, although this increase was insignificant.

The cell responses to different crosslinked PCLTA networks also provided the criteria of selecting biomaterials with sufficient crosslinking time to ensure appropriate crosslinking density and mechanical properties. PCLTAs were proven to have high crosslinking efficiency under UV exposure for only 5–10 min, and therefore, could be fabricated into two-dimensional substrates and three-dimensional scaffolds with better precision and controllability to fulfill different requirements for tissue engineering applications.

## 3. Materials and Methods

### 3.1. Photo-Crosslinking and Characterization of PCLTAs

**Figure 8:**
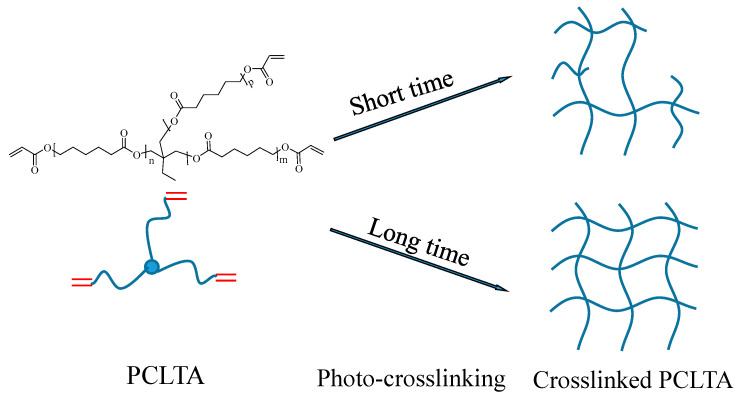
Scheme of the crosslinked poly(ε-caprolactone) acrylates (PCLTAs) with different crosslinking time.

Three PCLTAs with names of PCLTA8k, 10k, and 20k used in this study were synthesized according to a previous report and had number-average molecular weights (M_n_) of 8460, 9750, and 20,020 g/mol, respectively [18]. The PCLTAs were crosslinked into networks under UV light (SB-100P, λ = 365 nm, Intensity: 4800 w/cm^2^) for different crosslinking times of 1, 3, 5, 10, and 20 min, as shown in Figure 8. The gel fraction and swelling ratio in CH_2_Cl_2_ of the crosslinked PCLTAs were determined according to a previous report [17]. Flat uncrosslinked PCLTA substrates (crosslinking time of 0) were prepared by compression and crystallization of the melt between two glass plates. Flat photo-crosslinked PCLTA substrates were soaked in acetone for two days to remove the sol fraction, dried in vacuum, and compressed between two glass plates. The thermal properties of the photo-crosslinked PCLTAs were performed using differential scanning calorimeter (DSC, Q2000, TA instruments). The samples were first heated from room temperature to 100 °C and then cooled to −90 °C, followed by heating again to 100 °C at a rate of 10 °C/min in a dry nitrogen atmosphere. Linear viscoelastic properties of photo-crosslinked PCLTA disks (8 mm × 0.5 mm, diameter × thickness) were measured on a strain-controlled rheometer (RDS-2, Rheometric Scientific) at 37 °C. The surface morphologies of the photo-crosslinked PCLTAs were detected using a multi-mode atomic force microscope (AFM) with a Nanoscope III control system (Veeco Instruments, Santa Barbara, CA, USA). A tapping mode over a scan area of 5 μm × 5 μm at a scan rate of 0.5 Hz was used. Root-mean-square surface roughness (*R*_rms_) was measured from the height images using the Nanoscope 7.30 software (Veeco Instruments, Santa Barbara, CA, USA).

### 3.2. In Vitro SMC Attachment and Proliferation

Photo-crosslinked PCLTA disks were sterilized in 70% ethanol solution and dried completely before cell studies. The primary aortic SMCs of Sprague-Dawley rats (Charles River, Wilmington, NC, USA), similar to humans’ biological characteristics, were cultured in a growth medium composed of Dulbecco’s modified eagle medium (DMEM; Gibco, Grand Island, NY, USA) supplemented with 10% fetal bovine serum (FBS; HyClone, Thermal Scientific, TX, USA) and 1% penicillin/streptomycin (Gibco, Grand Island, NY, USA) in an incubator with 5% CO_2_ and 95% relative humidity at 37 °C. SMCs were seeded onto PCLTA disks (10 × 0.5 mm, diameter × thickness) at a density of 15,000 cells/cm^2^ and cultured for 4 h, 1, 2, and 4 days. A colorimetric cell metabolic assay (CellTiter 96 Aqueous One Solution, Promega, Madison, WI, USA) was performed in each well to determine the number of attached cells, which was correlated with the UV absorbance of the solution at 490 nm measured on the microplate reader (SpectraMax Plus 384, Molecular Devices, Sunnyvale, CA, USA). Cell numbers were then quantified using the standard curve that was constructed using known cell numbers. SMCs attached on PCLTA disks were washed with phosphate buffered saline (PBS), fixed in 4% paraformaldehyde (PFA, Electron Microscopy Science, PA, USA) solution for 10 min at room temperature, washed with PBS twice, and then permeabilized with 0.1% Triton X-100 at room temperature for another 10 min. The cytoplasm was then stained using rhodamine-phalloidin (RP, Cytoskeleton Inc., Denver, CO, USA) for 1 h at 37 °C, and cell nuclei were stained using 4′,6-diamidino-2-phenylindole (DAPI) at room temperature. Then, the cells were photographed using an Axiovert 25 light microscope (Carl Zeiss, Göttingen, Germany). From the cell images, cell area was determined on more than 50 non-overlapping cells at day 1 by using the ImageJ software (National Institutes of Health, Bethesda, MD, USA).

### 3.3. Focal Adhesions (FAs)

After day 1 post-seeding, SMCs attached on the PCLTA disks with crosslinking times of 0 and 3 min were washed with PBS, fixed in 4% PFA solution, washed with PBS three times, and permeabilized with 0.1% Triton X-100 at room temperature for 10 min. Then, the polymer disks with attached SMCs were incubated in 1% bovine serum albumin (BSA)/PBS at 37 °C for 30 min to reduce the background. After being washed with PBS three times, the polymer diskes with cells were incubated in monoclonal mouse antibody against vinculin (1:1000 in 1% PBS; Sigma, St. Louis, MO, USA) at room temperature for 2 h, and then washed with PBS three times again. Then, the samples were cultured with goat anti-mouse IgG secondary antibody (1:200 in 1% PBS; Sigma) in the dark at room temperature for 2 h. The samples were also stained using RP at 37 °C for another 1 h. The FAs were photographed using a Leica DM6000B fluorescent confocal microscope. The density, area, and circularity (defined as 4π × area/perimeter^2^) of FAs were measured and averaged from 15 non-overlapping cells using ImageJ.

### 3.4. Statistical Analysis

The statistical difference among varied experimental groups was analyzed using Kruskal-Wallis with Mann–Whitney post hoc test. Any two data groups with p-value calculated lower than 0.05 were marked with a significant difference.

## 4. Conclusions

UV light have been applied to prepare cross-linkable PCLTA. The influences of crosslinking time on cellular behaviors of PCLTAs with different molecular weight were studied. A lower molecular weight and a longer crosslinking time resulted in a higher gel fraction, a lower swelling ratio, and a higher stiffness. Photo-crosslinking of PCLTAs largely was accomplished in the first 5 min of UV exposure. SMCs were cultured on these photo-crosslinked PCLTAs. At the same crosslinking time, PCLTA with a higher molecular weight could be crosslinked into substrates better supporting SMC attachment and proliferation. With the same molecular weight, better SMC attachment, proliferation, spreading, and focal adhesion were found on the photo-crosslinked PCLTA substrates at a longer crosslinking time. The increments in SMC attachment and proliferation were no longer significant after the crosslinking time was longer than 10 min. Our photo-crosslinked PCLTA would be used to fabricate 3D scaffolds, demonstrating their potentials as biomaterials for diverse tissue-engineering applications.

## Figures and Tables

**Table 1:** Thermal properties of the crosslinked PCLTAs with different crosslinking time.

Crosslinking Time (min)	T_m_ (°C)			H_m_ (J/g)			χ_c_ (%)		
	8k	10k	20k	8k	10k	20k	8k	10k	20k
0	49.4	53.1	54.9	65.8	67.4	71.4	48.8	50.0	52.9
1	41.4	44.0	51.3	52.2	51.4	60.7	38.7	38.0	45.0
3	39.6	43.5	50.8	49.5	51.2	57.6	36.6	37.9	42.7
5	39.5	43.3	50.9	49.0	51.0	57.2	36.3	37.8	42.4
10	39.1	42.8	50.9	48.0	46.1	58.0	35.6	34.2	43.0
20	39.0	42.7	50.7	42.5	45.5	56.5	31.5	33.7	41.9
